# A Comparison of Particulate Matter from Biomass-Burning Rural and Non-Biomass-Burning Urban Households in Northeastern China

**DOI:** 10.1289/ehp.10622

**Published:** 2008-03-24

**Authors:** Ruoting Jiang, Michelle L. Bell

**Affiliations:** 1 Department of Civil and Environmental Engineering, Stanford University, Stanford, California, USA; 2 School of Forestry and Environmental Studies, Yale University, New Haven, Connecticut, USA

**Keywords:** biomass fuels, China, exposure assessment, household energy, indoor air pollution, particulate matter, PM_10_, PM_2.5_, rural health

## Abstract

**Background:**

Biomass fuel is the primary source of domestic fuel in much of rural China. Previous studies have not characterized particle exposure through time–activity diaries or personal monitoring in mainland China.

**Objectives:**

In this study we characterized indoor and personal particle exposure in six households in northeastern China (three urban, three rural) and explored differences by location, cooking status, activity, and fuel type. Rural homes used biomass. Urban homes used a combination of electricity and natural gas.

**Methods:**

Stationary monitors measured hourly indoor particulate matter (PM) with an aerodynamic diameter ≤ 10 μm (PM_10_) for rural and urban kitchens, urban sitting rooms, and outdoors. Personal monitors for PM with an aerodynamic diameter ≤ 2.5 μm (PM_2.5_) were employed for 10 participants. Time–activity patterns in 30-min intervals were recorded by researchers for each participant.

**Results:**

Stationary monitoring results indicate that rural kitchen PM_10_ levels are three times higher than those in urban kitchens during cooking. PM_10_ was 6.1 times higher during cooking periods than during noncooking periods for rural kitchens. Personal PM_2.5_ levels for rural cooks were 2.8–3.6 times higher than for all other participant categories. The highest PM_2.5_ exposures occurred during cooking periods for urban and rural cooks. However, rural cooks had 5.4 times higher PM_2.5_ levels during cooking than did urban cooks. Rural cooks spent 2.5 times more hours per day cooking than did their urban counterparts.

**Conclusions:**

These findings indicate that biomass burning for cooking contributes substantially to indoor particulate levels and that this exposure is particularly elevated for cooks. Second-by-second personal PM_2.5_ exposures revealed differences in exposures by population group and strong temporal heterogeneity that would be obscured by aggregate metrics.

About half the world’s population relies on biomass fuels as the primary domestic energy source (Smith et al. 2004). In rural China, biomass fuels account for about 80% of domestic energy (World Resources Institute 1998). Biomass combustion results in severe indoor air pollution, especially particulate matter (PM). Exposure to PM has been associated with increased risk for a suite of negative health outcomes, such as acute respiratory infection, chronic respiratory disease, and mortality (U.S. Environmental Protection Agency 2004).

The Chinese population suffers a high health burden from lung diseases, and respiratory disease is the primary cause of death in rural China (Schmidt 2002). Indoor PM from biomass fuels is one of the most serious yet least studied environmental health problems in China. In fact, little is known about human exposure to indoor PM in China and how different populations may be affected. Data are particularly lacking for rural China (Schmidt 2002). Several studies conducted in other parts of the world have investigated exposures to indoor air pollution from biomass fuels, finding that exposure patterns differed by sex, location in the home, and activity patterns (e.g., cooking vs. noncooking) (Balakrishnan et al. 2002; Ezzati et al. 2000).

Indoor air pollution in China has been explored in several previous studies. Respirable particles [RPM; PM with a median aerodynamic diameter ≤ 4 μm (PM_4_)], carbon monoxide, and sulfur dioxide were measured using stationary monitors at the household level in four Chinese provinces. The two provinces using biomass as the primary fuel had the highest PM_4_ concentrations (Jin et al. 2005). He et al. (2005) monitored multiple pollutants (PM_4_, CO, SO_2_, fluoride, and arsenic) at four points inside homes consuming coals and/or biomass fuels in the Guizhou and Shaanxi provinces. PM_4_ was higher in Guizhou households than in those in Shaanxi because of the fuel and stove combination (i.e., biomass fuel instead of coal, traditional stove instead of improved stove). Wang et al. (2006) investigated PM with an aerodynamic diameter ≤ 10 μm (PM_10_), PM with an aerodynamic diameter ≤ 2.5 μm (PM_2.5_), and 18 PM_2.5_ chemical components in four hospitals and adjacent outdoor environments in Guangzhou, China. Indoor PM_2.5_ levels in the hospitals were significantly higher than the U.S. Environmental Protection Agency ambient PM_2.5_ standard.

Additional summaries of research on exposure to indoor air pollution in China are provided elsewhere (Mestl et al. 2007a; Sinton et al. 1995). Despite these important studies, several unanswered questions remain. Specifically, estimates of indoor PM exposure typically were based on stationary monitors, often in combination with daily activity dairies, rather than continuous personal PM monitoring. Time–activity patterns were recorded by participants rather than researchers, introducing potential bias. Published studies do not include time–activity data for mainland China. In addition, monitoring generally applied exposure metrics of daily or hourly values and therefore has been unable to illuminate heterogeneity in exposure at smaller time scales. Finally, with the notable exception of Wang et al. (2006), most studies focused on PM_10_ or PM_4_ rather than PM_2.5_, although PM_2.5_ appears to be more closely linked to adverse health effects.

In this study we investigated indoor PM_10_ and PM_2.5_ levels in northeastern China using stationary and personal monitoring and time–activity diaries generated by direct observation. We compared exposures for cooks and noncooks, indoor and outdoor levels, urban and rural homes, and fuel type. Personal monitoring data include second-by-second measurements, allowing analysis of variation at small time scales. To the best of our knowledge, this is the first study to employ personal PM_2.5_ monitoring to assess individual exposures in China. In addition, we believe this study to be one of the first in mainland China to collect time–activity data.

## Materials and Methods

### Research location and sampling periods

**Figure 1:**
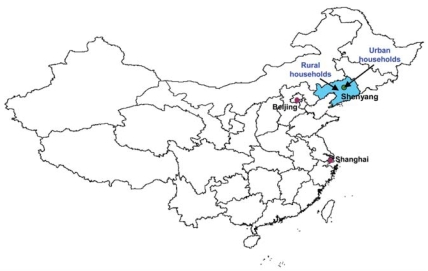
Map of study area showing Shenyang City (green circle), the capital of Liaoning Province (blue), China. Arrows note the approximate locations of the urban households (in Shenyang city) and rural households (in Liaozhong County). Liaozhong County is approximately 69 km southwest of Shenyang City.

This study was conducted in six households of Shenyang, the capital city of Liaoning Province in northeastern China (Figure 1). Three households were located in Shenyang rural areas, and three in Shenyang metropolitan areas. Household selection was based on feasibility and guidance of local environmental and governmental agencies.

Exposure analysis included *a*) stationary indoor and outdoor PM_10_ monitors, *b*) personal PM_2.5_ monitors, and *c*) time–activity dairies for study participants. Sampling was conducted within 25 May to 10 August 2006. During this time of year, biomass burning is used just for cooking, whereas in other time periods biomass is used for both cooking and heating. Thus, our results can be interpreted as isolating the impact of biomass burning for cooking. Stationary monitoring occurred over 5 consecutive days. In rural homes, stationary monitors were used to assess hourly PM_10_ levels in kitchens, and a single outdoor monitor at a rural home was used to measure ambient PM_10_. For urban homes, stationary monitors assessed hourly PM_10_ levels in kitchens and sitting rooms.

Adult household residents were surveyed to determine whether each person was a primary cook for the household and the time spent at home per day. All cooks and the noncooks spending most of their time at home were requested to participate in the personal monitoring and time–activity diary portion of the study. Ten of the 18 adult residents participated. Verbal consent to participate in the study was obtained from each participant. Personal monitors estimated continuous PM_2.5_ exposure with second-by-second resolution over 3 consecutive days for each participant. During the 3 days coinciding with personal monitoring, researchers kept time–activity dairies for each participant. Table 1 provides the sampling periods for stationary and personal monitoring for each household. The sampling period covers weekends and weekdays, although the work and activity patterns of this population are similar across days.

### Stationary PM_10_ monitoring

Stationary PM_10_ monitors were placed in 10 locations: three urban kitchens, three urban sitting rooms, three rural kitchens, and an outdoor rural location. Hourly concentrations were measured using P-5L_2_C Digital Dust Indicators manufactured by Beijing Binta Green Technology Co., Ltd. (Beijing, China). These devices determine relative PM_10_ concentrations based on the intensity of light scattered by particles passing through an illumination chamber. This intensity is measured by a photo multiplier tube located at a 90° angle to the light source and converted to pulses, which are indicated in count per minute values that are then converted to mass PM_10_ concentrations (Beijing Binta Green Technology Co. 2007).

For rural households, PM_10_ levels in indoor kitchens were measured approximately 1 m from the stove. Outdoor PM_10_ levels were measured in the yard of rural household 2 approximately 0.8 m from the house. Rural household 2 was 80 m and 50 m from the other rural households. For urban homes, monitors were approximately 1 m from the gas stove in kitchens and in the center of sitting rooms. All stationary monitors were placed on a flat surface at a height of approximately 0.6 m. Other criteria for choosing the sampling positions were access to electricity to power the monitors, the safety and stabilization of equipment, and avoidance of interference with household activities. During sampling periods, PM_10_ monitors operated continuously each day for approximately 14 hr from 0530 to 1930 hours for rural households and for approximately 10 hr from 0830 to 1830 hours for urban households.

Urban PM_10_ levels were obtained from the Shenyang Environmental Bureau, which measures 24-hr PM_10_ at eight locations across the city (Er Mao, Tai Yuan Street, Xiao He Yan, Wen Yi Road, North Mausoleum, Cannon School, Zhang Shi, and Dong Ruan). Daily PM_10_ levels were calculated from publicly available air pollution index values (Shenyang Environmental Bureau 2007) based on the guidelines provided by the China National Environmental Monitoring Center (2007).

The Shenyang Environmental Bureau used automated continuous sampling methods for PM_10_, which is measured using tapered element oscillating microbalance technology and reported at averaging times of 24 hr (Zhou, Shenyang Environmental Bureau Monitoring Center, personal communication).

### Personal PM_2.5_ monitoring

Personal PM_2.5_ exposures for 10 participants were measured using model AM510 SidePak personal aerosol monitors (aTSI Inc. 2006a). These monitors use light-scattering technology to determine mass concentration in real time at 1-sec intervals. An aerosol sample is drawn into the sensing chamber in a continuous stream. A laser illuminates one section of the aerosol stream. A lens at 90° to both the aerosol stream and laser beam collects light scattered by particles and focuses it onto a photo detector. The detection circuitry converts the light into voltage, which is proportional to the mass concentrations of aerosols. The voltage is read by the processor and multiplied by an internal calibration constant to provide mass concentration (bTSI Inc. 2006b).

**Figure 2:**
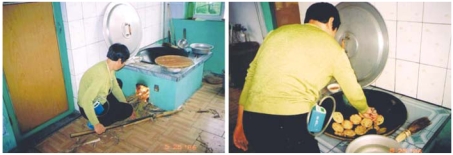
Study participant with the personal monitor in a rural setting: using biomass to fuel the stove (*A*) and cooking (*B*).

These lightweight monitors were equipped with personal pumps and attached to the belts of participants. Tubing connected the inlet of each monitor to the individual’s collar to sample the breathing zone (Figure 2). Each individual was instructed to carry the monitor indoors and outdoors during waking hours (~ 15 hr/day) throughout the sampling period, except while sleeping, showering, and using the restroom. Participants were instructed to place the monitors at approximately 1–1.5 m above the ground surface (i.e., close to the breathing zone) when the monitor could not be carried.

### Time–activity diaries

Throughout the personal monitoring sampling periods, the principal researcher (R.J.) and a research assistant maintained written 24-hr time–activity diaries for each participant (Table 1). Whereas most previous research had subjects record their own activities, this study applied direct observation in real time to eliminate recall bias and ensure uniform treatment across participants. Time–activity diaries recorded participants’ location (outdoors vs. indoors) and activities in 30-min intervals. Activities were divided into the following categories: cooking (e.g., preparation for cooking, such as cleaning stove, lighting, and tending fire), sleeping at nighttime, eating, socializing (e.g., conversing), relaxing (e.g., watching television, playing with children, napping during daytime), cleaning, and other (e.g., outside, all other activities not listed above). Researchers also noted housing characteristics and fuel type for cooking in each home.

### Data analysis

We compared stationary PM_10_ measurements between rural and urban households, kitchens, and sitting rooms in urban households, and indoor and outdoor levels. We examined personal PM_2.5_ exposures by activity pattern (e.g., cooking vs. noncooking) and participant group (e.g., urban cook vs. rural cook). We applied descriptive statistics, Pearson’s correlation coefficients, linear regression analysis, and analysis of variance (ANOVA). Minitab statistical software (Minitab Inc., State College, PA, USA), TrakPro data analysis software, version 3.41 (TSI Inc., Shoreview, MN, USA) and the R statistical package, version 2.4.1 (http://www.r-project.org), were used.

We analyzed the relationship between hourly PM_10_ levels in urban kitchens and sitting rooms with linear regression as follows:


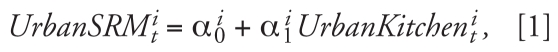


where *UrbanSRM_t_^i^* is the PM_10_ concentration for hour *t* for the sitting room of urban house-hold *i*, *UrbanKitchen_t_^i^* is the PM_10_ concentration for hour *t* for the kitchen of urban household *i*, and α_0_*^i^* α_1_*^i^* are the regression coefficients for the relationship between PM_10_ kitchen and sitting room levels of household *i*.

We examined the relationship between rural indoor and outdoor hourly PM_10_ levels as follows:


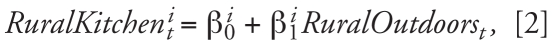


where *RuralKitchen_t_^i^* is the PM_10_ concentration for hour *t* for the kitchen of rural household *i*, *RuralOutdoors_t_* is the PM_10_ concentration for hour *t* for the rural outdoor monitor located at rural household 2, and β_0_*^i^* β_1_*^i^* are the regression coefficients for the relationship between PM_10_ levels outdoors and in the kitchen of household *i*.

The above regression analysis was performed separately for each rural household for cooking times, noncooking times, and the entire study period. A cooking episode was designated for any hour for which cooking took place in a time–activity diary for that household. A single outdoor monitor, located outside rural household 2, was used to estimate representative ambient concentrations for the rural community.

## Results

### Housing and participant characteristics

All rural homes in the study were one-story houses with large yards for crop cultivating and animal husbandry. All the urban homes were apartments located in central Shenyang, two on the eleventh floor and one on the fourth floor. For all households, kitchens and living areas were separate. The rural homes used biomass fuels (corn) for cooking, whereas the urban homes used a combination of natural gas and electricity. Exhaust fans existed in all the urban kitchens; none were in the rural kitchens.

Ten household residents participated in the personal monitoring and time–activity diary portions of the study: three female cooks, a female noncook, and a male noncook in urban households and three female cooks, a female noncook, and a male noncook in rural households. All subjects were adults, with an average age of 61 years (range, 19–85 years).

### Stationary PM_10_ monitoring results

**Figure 3:**
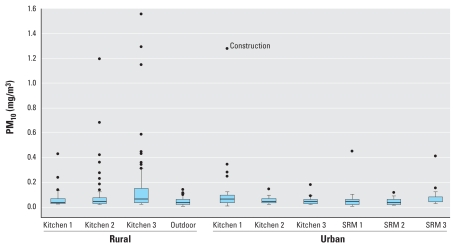
Box plots of hourly measurements from PM_10_ stationary monitors (mg/m^3^). SRM, sitting room. An outlier value for urban kitchen 1 took place during indoor construction.

Table 2 summarizes PM_10_ levels based on hourly measurements from stationary monitors at various indoor locations on average across specific groups of households (rural or urban) and the outdoor locations. Urban outdoor levels, likely resulting from transportation networks and growing urbanization, exceeded rural outdoor levels and urban indoor levels. Figure 3 provides box plots of the hourly PM_10_ stationary monitors. An outlier value of 1287.0 μg/m^3^ occurred in urban kitchen 1 during a period of indoor construction. PM_10_ levels in rural kitchens were 64% higher on average than in urban kitchens and 2.5 times higher than outdoor levels. Urban kitchen and sitting rooms had similar concentrations, which were lower than the urban outdoors concentration. Rural kitchen PM_10_ levels had the highest recorded levels and exhibited the largest variability.

### Comparison of PM_10_ levels in urban and rural kitchens

Kitchen measurements were divided into cooking and noncooking times to explore how different fuel types and kitchen designs affect PM_10_ levels. A cooking time was defined as a period with “cooking” in the activity diary for at least one participant in the household. Table 3 shows PM_10_ concentrations in kitchens based on stationary hourly measurements, divided by cooking and noncooking times, averaged by home type (urban or rural). During cooking, kitchen PM_10_ levels in rural households were on average 3.0 times higher than in urban households (one-way ANOVA, *p*< 0.05), whereas the PM_10_ levels for urban and rural households during noncooking times are not statistically different (*p* > 0.05).

### Comparison of kitchen and sitting room PM_10_ levels for urban households

PM_10_ levels in urban sitting rooms were similar to but lower than concentrations in urban kitchens (Table 2). The relationship between PM_10_ levels in these two types of areas was analyzed with correlation coefficients and with linear regression for each urban household (Table 4). Regression analysis results are presented as the percent change in the urban sitting room PM_10_ level per 10 μg/m^3^ increase in the urban kitchen PM_10_ levels and 95% confidence interval (CI), evaluated at the mean sitting room level for that household. Findings indicate that in each household, PM_10_ levels in sitting rooms and kitchens are strongly related.

### Comparison of indoor and outdoor PM_10_ levels for rural households

Table 5 shows correlation coefficients comparing the hourly PM_10_ measurements from the rural kitchens to the rural outdoor monitor, stratified by cooking and noncooking periods. Table 5 also presents results from the regression analysis for each household, stratified by cooking and non-cooking periods. Although the outdoor monitor is located near rural home 2, this home does not exhibit the strongest relationship between indoor and outdoor PM_10_ levels. Rural home 1 had higher indoor (kitchen) PM_10_ levels during periods of higher outdoor levels (*p* < 0.05), yet no relationship was observed between outdoor and kitchen levels for the other homes, based on data for the entire study period. No relationship was observed between outdoor and kitchen PM_10_ levels during cooking periods. However, during noncooking periods, rural kitchen and outdoor PM_10_ levels were positively associated (statistically significant for rural homes 1 and 3).

### Time–activity patterns

**Figure 4:**
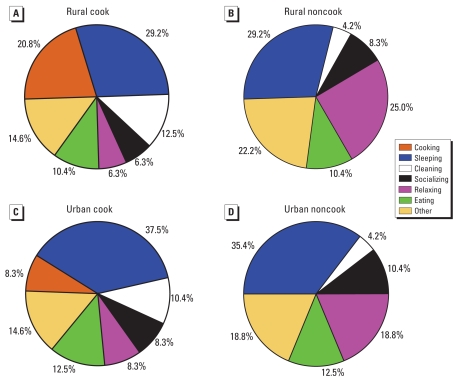
Time–activity budgets of three rural cooks (*A*), two rural noncooks (*B*), three urban cooks (*C*), and two urban noncooks (*D*).

Figure 4 describes the participants’ time–activity budgets based on 24-hr assessments for various participant categories: rural cooks (*n* = 3), rural noncooks (*n* = 2), urban cooks (*n* = 3), and urban non-cooks (*n* = 2). Generally, cooks spent 8.3–20.8% of the total time cooking. Rural cooks averaged 5 hr/day cooking, versus 2 hr/day for urban cooks. Cooking took place three times per day for each rural household, twice per day for urban homes 1 and 2, and once per day for urban home 3. The average time for each cooking event was 1.7 hr for rural homes and 1.2 hr for urban homes.

### Comparison of PM_2.5_ levels from personal monitoring by participant and activity

Table 6 shows average PM_2.5_ exposure by participant category (cook or noncook) and urban or rural designation, and by activity, as measured during waking hours (approximately a 16-hr period). Cooking periods had higher personal PM_2.5_ exposures than all other activity categories for cooks, but especially for rural cooks. The various noncooking activities had similar PM_2.5_ levels. Personal PM_2.5_ exposure for rural cooks was 3.3 times higher than for urban cooks (one-way ANOVA, *p* < 0.05) and 3.6 times higher than for rural noncooks (*p* < 0.05).

**Figure 5:**
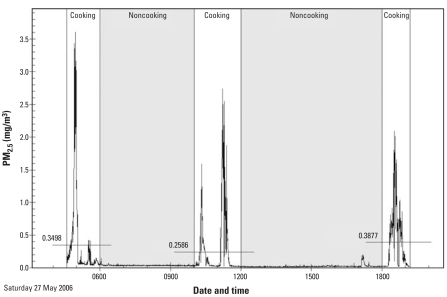
Variations of personal PM_2.5_ in a rural cook, based on second-by-second concentrations. Horizontal lines reflect the average for each cooking period.

Variability in PM_2.5_ exposures was investigated using the second-by-second measurements from personal monitors. Cooking times exhibited more heterogeneity in PM_2.5_ levels than did any noncooking activity for urban or rural cooks, particularly for rural cooks (Table 6). Second-by-second PM_2.5_ personal exposures for a rural cook over a 1-day period are shown in Figure 5, demontrating the higher PM_2.5_ levels and variability during cooking periods. During the three cooking periods, mean PM_2.5_ concentrations for this participant were 349.8, 256.8, and 387.7 μg/m^3^, compared with 37.5 and 22.9 μg/m^3^ during the noncooking periods. The standard deviations of PM_2.5_ during cooking periods were 661.5, 463.2, and 464.3 μg/m^3^, compared with 7.2 and 17.0 μg/m^3^ during noncooking times.

## Discussion

Our results indicate higher PM levels for households using biomass compared with those using cleaner fuels, cooks compared with noncooks, and cooking times compared with noncooking periods for households using biomass in northeastern China. Although the generalizability of our results is limited by the small sample size, these findings confirm similar results identified by studies in other regions (Ezzati and Kammen 2002) and add to the growing body of evidence that biomass fuels can result in highly elevated indoor air pollution levels, which in turn can contribute to adverse health effects.

Unique aspects of our study, in addition to the location, include the use of personal PM_2.5_ monitoring at second-by-second resolution, allowing analysis of heterogeneity at small time scales. The personal exposure monitoring data exhibit variation that would be obscured by the use of more aggregate measures, especially during cooking periods. Another unique aspect is the use of researchers rather than participants to record time–activity dairies in real time, which avoids recall bias and encourages consistency. To the best of our knowledge, this study is the first to employ personal PM_2.5_ monitoring to assess PM exposures in China, and the first study in mainland China to collect time–activity data.

Measured concentrations for rural kitchens were lower than PM kitchen levels of rural households burning biomass as measured in other studies (Table 7). The lower levels observed in this study may be related to the good conditions of the stoves, because all stoves in the participating rural households were refurbished within the 5 years preceding the study. Also, this study was conducted at the end of May, which is not a major season of rural biomass consumption. Thus, PM levels are likely to be even higher during the winter season, when biomass is used for heating as well as cooking in rural households. Our findings show that high PM levels are experienced even under conditions of refurbished stoves in the nonheating season.

PM_10_ levels in rural kitchens were 64% above those in urban kitchens and 2.5 times higher than outdoor concentrations, consistent with earlier work finding higher PM levels in rural kitchens compared with the ambient environment (Table 8). Higher pollution levels in kitchens compared with other rooms for households using biomass fuel have been documented in other regions. In a study of rural homes using biomass in Mpala Ranch, central Kenya, PM_10_ concentrations were 3.5–7.5 times higher in areas close to the stove compared with other areas (Ezzati et al. 2000). In Andra Pradesh, India, PM_4_ levels were 1.5–2 times higher in kitchens than in living rooms in rural households burning biomass (Balakrishnan et al. 2004).

We found that urban households, which used cleaner fuels (natural gas), had significantly lower indoor and personal PM levels than did rural households, which used biomass. Rural kitchens were equipped with low-efficiency chimneys compared with the highly efficient exhaust fans in urban kitchens. This conclusion is supported by earlier findings, such as those of Röllin et al. (2004), who reported elevated indoor RPM levels in non-electrified dwellings relying on biomass as domestic energy, compared with homes using electricity or a mix of electricity and biomass fuels for cooking, in rural South Africa. In Tamil Nadu, India, cooks using biomass fuels experienced higher indoor PM_4_ levels than did cooks using clean fuels such as kerosene or gas (Balakrishnan et al. 2002).

During cooking, rural cooks had personal PM_2.5_ exposure 5.4 times higher than urban cooks. Whereas urban cooks and noncooks had similar personal exposures, rural cooks’ PM_2.5_ exposure was 3.6 times higher than that of rural noncooks. Other studies revealing different personal PM exposure based on cooking fuels include research of rural households in southern India, which found concentrations of respiratory particles for cooks using wood and crop residue to be 2.6 times higher than for noncooks and 2.8 times higher than for cooks using clean fuel (Balakrishnan et al. 2002). In rural Kenya, adult women had the highest PM_10_ levels (4,898 μg/m^3^), and both young and adult women had higher exposures than did their male counterparts. For cooks, the high-intensity emission episodes accounted for 31–61% of total exposure (Ezzati et al. 2000). In Maputo, Mozambique, biomass users were exposed to significantly higher PM levels during cooking (540–1,200 μg/m^3^) than were users of liquefied petroleum gas and electricity (200–380 μg/m^3^) (Ellegård 1996). A study of three households in Highland Guatemala showed that personal exposures of mothers and children using biomass are higher than exposures of those using natural gas (Naeher et al. 2000).

Time–activity diaries recorded by researchers in 30-min intervals showed that rural cooks spent 2.5 times more hours per day cooking than did urban cooks, with a higher frequency and length of cooking events. One reason for this difference is that rural cooks need to clear the stove, fetch biomass, and light biomass before their cooking activities, whereas the urban cooks in this study used the more efficient and less time-consuming fuels natural gas and/or electricity. Jin et al. (2006) reported that women in four Chinese provinces spend approximately 2–3 hr/day cooking, whereas in our study, rural cooks spent 5 hr/day cooking and urban cooks 2 hr/day.

Other studies evaluated time–activity diary data according to time spent in various microenvironments (e.g., kitchen, living room). In Tami Nadu, India, women cooks spent 6.76 hr/day in the kitchen, compared with 0.76 hr for women not involved in cooking (Balakrishnan et al. 2002). In central Kenya, some household members, primarily cooks, spent more time in the kitchen close to the fire when pollution concentrations were high, while other household members were outside the kitchen (Ezzati and Kammen 2001). Similar results were found in Kenya (Ezzati et al. 2000), rural Bolivia (Albalak et al. 1999), Andhra Pradesh, India (Balakrishnan et al. 2004), and the Shanxi Province of China (Mestl et al. 2006).

The higher PM exposures from biomass burning in this study are likely associated with adverse health effects. A review of health studies researching the burning of coal and biomass fuels in Chinese households found evidence of a severe health burden, including respiratory disease and impaired lung function (Zhang and Smith 2007). Several studies have linked fuel and stove type to health effects, such as increased risk of cataracts for women cooks in Nepal and India (Pokhrel et al. 2005), asthma symptoms in children in homes with open fires compared with children in homes with improved stoves with chimneys (Schei et al. 2004), more eye discomfort for women using open fires compared with those using improved stoves (Díaz et al. 2007), and higher frequency of cytogenetic alterations in blood lymphocytes for users of biomass fuels compared with women using liquefied petroleum gas (Musthapa et al. 2004). In Tanzania, acute respiratory illness was more prevalent for cooks and children < 5 years of age compared with men and noncooking women, likely because of differences in biomass burning exposure (Kilabuko et al. 2007). Risk of lung cancer was lower for residents of houses with separate kitchens or improved air circulation in Guangzhou, China (Liu et al. 1993). Mestl et al. (2006) estimated that lower household solid fuel use in Shanxi Province would reduce childhood asthma and adult respiratory disease.

Indoor air pollution is responsible for an estimated 4–5% of deaths in developing countries (Smith and Mehta 2003) and 4–6% of the burden of disease (Smith 2000). Approximately 3.5 million deaths per year in China are attributable to indoor air pollution (Mestl et al. 2007b). Programs to reduce these health responses include installation of improved stove types that increase energy efficiency, substitution of cleaner fuels, and new technologies such as biodigesters (Edwards et al. 2004; Zhang and Smith 2007). Still, the dominance of biomass fuels for energy use in rural Chinese households is anticipated to continue (Li et al. 2007).

Our findings indicate that the use of biomass for cooking by rural households greatly elevates PM exposures, particularly for those performing the cooking, and emphasizes the need for additional research on alternative fuels and kitchen and stove designs, as well as on the health burden from this pollution. Further, research is needed to investigate the intersection between policies aimed at improving indoor air quality and those intended to lower emissions of greenhouse gases (Smith 2002).

## Figures and Tables

**Table 1:** Sampling periods for exposure analysis.

Type and location of monitoring	Sampling period
Stationary monitoring (PM_10_)	
Urban kitchen 1 and urban sitting room 1	2–6 Aug 2006
Urban kitchen 2 and urban sitting room 2	5–9 Aug 2006
Urban kitchen 3 and urban sitting room 3	7–11 Aug 2006
Urban outdoors^a^	2–11 Aug 2006
Rural kitchen 1	25–29 May 2006
Rural kitchen 2	26–30 May 2006
Rural kitchen 3	26–30 May 2006
Rural outdoors	25–30 May 2006
Personal monitoring (PM_2.5_) and time–activity dairies	
Urban cook 1 and urban noncook 1	2–4 Aug 2006
Urban cook 2	5–7 Aug 2006
Urban cook 3 and urban noncook 3	8–10 Aug 2006
Rural cook 1 and rural noncook 1	25–27 May 2006
Rural cook 2	26–28 May 2006
Rural cook 3 and rural noncook 3	28–30 May 2006

The three urban households are designated urban 1, urban 2, and urban 3, and likewise for rural households. Urban cook 1 corresponds to a participant in household urban 1, etc.

a Urban outdoor PM_10_ levels were obtained from the Shenyang Environmental Bureau.

**Table 2:** PM_10_ levels from stationary monitors (μg/m^3^).

Location	Mean ± SD	Median	Minimum–maximum
Indoors			
Rural kitchens	100.6 ± 203.1	45.00	14.00–1571.0
Urban kitchens	61.34 ± 111.8 52.77 ± 44.08^a^	43.00 43.00^a^	2.00–1287.0 2.00–335.0^a^
Urban sitting rooms	48.46 ± 51.97	36.00	0.00–448.0
Outdoors			
Rural	40.23 ± 26.50	32.00	2.00–133.0
Urban^b^	89.20 ± 17.84	80.00	74.00–126.0^b^

Three households each were used to estimate concentrations for rural kitchens, urban kitchens, and urban sitting rooms. Median, minimum, and maximum refer to hourly levels. The minimum and maximum represent the lowest and highest hourly levels recorded in any household. Urban indoor values are based on 10-hr sampling periods, and rural values, on 14-hr sampling periods.

a Excludes outlier value from indoor construction for urban kitchen 1.

b Mean urban outdoor levels were based on 24-hr averages; minimum and maximum outdoor levels reflect daily values.

**Table 3:** PM_10_ levels for urban and rural kitchens, stratified by cooking and noncooking periods (μg/m^3^).

	Total study period		Cooking times		Noncooking times	
Household type	No. of hours	PM_10_ (mean ± SD)	No. of hours	PM_10_ (mean ± SD)	No. of hours	PM_10_ (mean ± SD)
Rural	190	100.6 ± 203.1	76	202.1 ± 293.6	114	33.01 ± 15.31
Urban	144 143^a^	61.34 ± 111.8 52.77 ± 44.08^a^	29	67.00 ± 32.58	115 114^a^	59.40 ± 123.8 48.62 ± 44.83^a^

a Excludes outlier value from indoor construction for urban kitchen 1.

**Table 4:** Relationship between hourly urban sitting room PM_10_ levels and urban kitchen PM_10_ levels.

Home designation	Correlation coefficient (*p*-value)	Percent change in urban sitting room PM_10_ per 10 μg/m^3^ increase in kitchen PM_10_ (95% CI)
Urban home 1	0.77 (0.051) 0.77 (< 0.001)^a^	1.79 (0.04–3.53) 14.40 (10.97–17.82)^a^
Urban home 2	0.86 (< 0.001)	24.27 (20.51–28.03)
Urban home 3	0.93 (< 0.001)	29.60 (25.32–33.88)

a Excludes outlier value from indoor construction for urban kitchen 1.

**Table 5:** Relationship between rural kitchen and rural outdoor PM_10_ levels.

	Correlation coefficients between rural kitchen and rural outdoor PM_10_ levels (*p*-value)			Percent increase in rural kitchen PM_10_ per 10 μg/m^3^ increase in rural outdoor PM_10_ levels, evaluated at the mean (95% CI)		
Home designation	Entire study period	Cooking times	Noncooking times	Entire study period	Cooking times	Noncooking times
Rural home 1	0.397 (0.004)	0.121 (0.633)	0.900 (< 0.001)	8.80 (3.10 to 14.50)	1.22 (−3.98 to 6.12)	23.58 (19.57 to 27.59)
Rural home 2	−0.043 (0.760)	−0.018 (0.936)	0.157 (0.407)	−4.16 (−30.76 to 22.43)	−1.29 (−32.10 to 29.52)	14.26 (−18.91 to 47.43)
Rural home 3	−0.057 (0.658)	−0.270 (0.183)	0.845 (< 0.001)	−4.40 (−23.74 to 14.96)	−11.98 (−29.09 to 5.13)	23.95 (18.86 to 29.04)

**Table 6:** Personal exposure PM_2.5_ levels (mean ± SD) by urban or rural designation, participant type, and activity based on second-by-second measurements (μg/m^3^).

	Rural cook (*n* = 3)	Rural noncook (*n* = 2)	Urban cook (*n* = 3)	Urban noncook (*n* = 2)
Cooking	487.9 ± 874.9	—	90.1 ± 120.9	—
Noncooking				
Cleaning	76.9 ± 58.2	73.8 ± 58.5	62.4 ± 29.5	83.5 ± 145.1
Socializing	51.3 ± 27.8	42.2 ± 31.1	52.5 ± 31.3	62.6 ± 30.3
Relaxing	39.5 ± 26.3	50.7 ± 37.8	48.7 ± 104.9	61.9 ± 36.2
Eating	86.9 ± 65.5	70.2 ± 64.8	60.7 ± 30.5	73.4 ± 44.4
Other	59.9 ± 64.1	65.8 ± 31.1	54.3 ± 32.4	72.5 ± 49.6
Total	201.5 ± 539.8	56.4 ± 51.1	61.7 ± 48.3	71.5 ± 72.9

**Table 7:** Comparison of the rural indoor kitchen or cooking room particulate levels based on stationary monitoring in this study and previous studies.

Location, study period (reference)	PM size	Type of fuel	Mean (μg/m^3^)	Notes
China				
Shenyang, China, May 2006 (this study)	PM_10_	Crop residue	Total: 100.6 Cooking times: 202.1	Based on ~14 hr/day (0530–1930) for 5 consecutive days 3 households
Jilan, China, Nov–Dec 2001, Feb–Mar 2003, 2004 (Fischer and Koshland 2007)	PM_4_	Multiple fuels: coal, biomass, gas, electricity	Daily average: 312 1-hr peak: 1,880	Based on 24-hr periods and 1-hr peak values 70 household-days 37 households
Gansu, China, Mar–Apr 2003, Dec 2003–Jan 2004 (Jin et al. 2005)	PM_4_	Wood and crop residue	Mar–Apr 2003: 518 Dec 2003–Jan 2004: 661	Based on 24-hr/day periods Mar–Apr 2003: 72 households with 1 day of measurement, 6 households with 4 days of measurement Dec 2003–Jan 2004: 17 households with 1 day of measurement, 6 households with 2–3 days of measurement
Guizhou, China, Mar–Apr 2003, Dec 2003–Jan 2004 (Jin et al. 2005)	PM_4_	Coal, wood, and crop residue	Mar–Apr 2003: 352 Dec 2003–Jan 2004: 301	Based on 24-hr/day periods Mar–Apr 2003: 76 households with 1 day of measurement, 7 households with 2–4 days of measurement Dec 2003–Jan 2004: 16 households with 1 day of measurement, 6 households with 2–3 days of measurement
Inner Mongolia, China, Dec 2003–Jan 2004 (Jin et al. 2005)	PM_4_	Wood and crop residue	718	Based on 24-hr/day periods 49 households with 1 day of measurement, 4 households with 3 days of measurement
Shaanxi, China, Mar–Apr 2003, Dec 2003–Jan 2004 (Jin et al. 2005)	PM_4_	50% coal, 50% wood and crop residue	Mar–Apr 2003: 187 Dec 2003–Jan 2004: 223	Based on 24-hr/day periods Mar–Apr 2003: 75 households with 1 day of measurement, 6 households with 4 days of measurement Dec 2003–Jan 2004: 18 households with 1 day of measurement, 6 households with 3 days of measurement
Guizhou, China, Jan 2003 (He et al. 2005)	PM_4_	Coal (3 households), coal and biomass (1 household)	1,944	Based on 24-hr/day periods for 4 consecutive days 4 households
Shaanxi, China, Feb 2003 (He et al. 2005)	PM_4_	Coal and biomass for cooking, coal for heating	205	Based on 24-hr/day periods for 4 consecutive days 4 households
Zhejiang, Hubei, and Shaanxi, China, Jun–Aug 2002, Dec 2002–Jan 2003 (Edwards et al. 2007)	PM_4_	Crop residues	Summer: 282.9 Winter: 456.4	Based on 24-hr periods 48 households for summer, 25 for winter
Bolivia				
Cantuyo, Jan 1994–Oct 1995 (Albalak et al. 1999)	PM_10_	Dung	1,830	Based on 6-hr periods (0500–1100)/day every 6 days for 3 consecutive weeks for each month of study period 12 households
India				
Pauri District, Garhwal Himalaya, northern India, Aug 1989–Jul 1990 (Saksena et al. 1992)	TSP	Wood	Cooking times: 5,600 Noncooking times: 820	Based on 15-hr periods 12 households
Tamil Nadu, southern India, Jul–Dec 1999 (Balakrishnan et al. 2002)	PM_4_	Wood and crop residue	Indoor kitchen without partitions: 1,442 Kitchen with partitions: 970	Based on 10- to 12-hr/day periods for 1–3 days Without partitions: 105 households With partitions: 68 households
Andhra Pradesh, southern India, Jan–May 2001 (Balakrishnan et al. 2004)	PM_4_	Wood Dung	Wood: 500 Dung: 732	Based on 22- to 24-hr/day sampling periods for 3–4 days Wood: 270 households Dung: 97 households
Guatemala				
Quetzaltenango, May–Nov 1993 (Naeher et al. 2000)	PM_2.5_ PM_10_ TSP	Wood	PM_2.5_: 527.9,^a^ 96.5^b^ PM_10_: 717.1,^a^ 186.3^b^ TSP: 835.8,^a^ 275.5^b^	Based on three 22-hr sampling periods 3 households
La Victoria, Jan 1999 (Bruce et al. 2004)	PM_3.5_	Wood and crop residue	1,019^a^ 351^b^	Based on one 24-hr measurement/ household 11 households with open fires, 5 with planchas
La Victoria, Dec 1998–Jul 1999 (Albalak et al. 2001)	PM_3.5_	Wood	1,560^a^ 280^b^	Based on 24-hr measurements taken 6 times at 1-month intervals 10 households of each stove type

TSP, total suspended particles.

a Traditional open fire stove.

b Improved plancha stove–equipped.

**Table 8:** Comparison of PM levels in rural kitchens and outdoor environments based on stationary monitoring in this study and previous studies.

Location (reference)	PM size	Type of fuel	Kitchen mean PM (μg/m^3^)	Outdoor mean PM (μg/m^3^)
Shenyang, China (this study)	PM_10_	Crop residue	100.6	40.23
Andhra Pradesh, southern India (Balakrishnan et al. 2004)	PM_4_	Wood Dung	500 732	87 99
Guizhou, China (He et al. 2005)	PM_4_	Coal and biomass	1,944–2,334	206
Shaanxi, China (He et al. 2005)	PM_4_	Coal and biomass	456	122
Cantuyo, Bolivia (Albalak et al. 1999)	PM_10_	Dung	3,690 (mean) 1,830 (geometric mean)	60 (mean) 50 (geometric mean)
Tanzania (Kilabuko et al. 2007)	PM_10_	Wood	656.2 (cooking times) 96.1 (noncooking times)	40.1
